# Enhancement of Fracture Toughness of Inner Liner Material for Type IV Hydrogen Storage Cylinders Based on Molecular Dynamics Method

**DOI:** 10.3390/ma18061363

**Published:** 2025-03-19

**Authors:** Bingyu Yang, Jinqi Luo, Yuan Wu, Zhenhan Yang, Jianping Zhao

**Affiliations:** 1 School of Mechanical and Power Engineering, Nanjing Tech University, Nanjing 211816, China; byyang0202@163.com (B.Y.); 18732457091@163.com (J.L.); 15255645044@163.com (Y.W.); yangzh20000316@163.com (Z.Y.); 2 Institute of Reliability Centered Manufacturing (IRCM), Nanjing Tech University, Nanjing 211816, China

**Keywords:** polyamide 6, high-density polyethylene, fracture toughness, molecular dynamics

## Abstract

To develop liner materials with improved toughness, this study combines molecular dynamics simulations and experimental testing to investigate the effect of different mass ratios (10/0, 7/3, 6/4, 4/6, 3/7, and 0/10) of high-density polyethylene (HDPE)/polyamide 6 (PA6) on their fracture toughness of the composites. The fracture toughness was quantitatively assessed using the J-integral method, while the material’s behavior in terms of crack propagation during tensile deformation was examined at the molecular level. The results reveal that as the HDPE mass ratio increases, the fracture toughness of the composites also gradually improves. Furthermore, the fracture toughness of four materials (PA6, 4HDPE/6PA6, 7HDPE/3PA6, and HDPE) was tested using the essential work of the fracture method. The trend observed in the simulation results was in agreement with the experimental results, validating the reliability of the molecular dynamics simulation.

## 1. Introduction

Hydrogen, as a highly efficient and environmentally friendly renewable energy source, offers a wide range of potential applications [1,2,3]. In terms of hydrogen storage [4], high-pressure gaseous hydrogen storage is currently one of the most widely used methods. High-pressure hydrogen storage tanks can be broadly classified into five types, with the first four having been commercialized [5]. Type I and II tanks have a high weight-to-storage ratio and are mainly used for stationary applications [6]. Type III tanks, with a metal liner, are prone to hydrogen embrittlement and fatigue failure under high-pressure hydrogen environments, leading to a shift in focus toward Type IV tanks with non-metallic liners [7,8]. With advantages such as lightweight structure, high hydrogen storage density, fast charging and discharging, and resistance to fatigue failure, Type IV tanks have become the primary choice for high-pressure hydrogen storage in many countries [9,10,11]. High-density polyethylene (HDPE) and polyamide (PA) are the two commonly used materials for the liner of Type IV hydrogen storage cylinders [12,13,14]. Due to its strong molecular polarity and hydrogen bonding interactions, polyamide exhibits lower hydrogen permeability compared to HDPE [15], with polyamide 6 (PA6) in particular demonstrating superior gas barrier properties. However, PA6 suffers from drawbacks such as high brittleness and poor dimensional stability [16,17,18], making it prone to cracking during service. These limitations hinder its performance as a liner material in practical applications. Consequently, modification of the pure material is required to enhance its toughness and meet the performance demands for use as a liner material.

Polymer blending represents an economical and promising method for a wide range of applications. The blending of multiple polymers can effectively enhance certain properties of the blends [19,20,21]. Tang et al. [22] investigated the impact of an ESI addition on the mechanical properties and morphology of PS/HDPE blends in a binary system. Their findings revealed that the strength of PS/HDPE/ESI composites decreased significantly as the HDPE content increased at a fixed level of the compatibilizer ESI. However, the inclusion of HDPE improved the tensile ductility and toughness of the composites. Wang et al. [18] developed PA6/HDPE composites with varying ratios through blending and observed that both the helium permeability coefficient and elongation at break of the composites gradually decreased as the PA6 content increased.

It is not uncommon for experimental studies to require a significant investment of time and resources. In light of the accelerated advancement of computer simulation technology, molecular dynamics (MD) simulation, as a supplementary methodology, has increasingly emerged as a pivotal instrument for investigating these intricate phenomena [23,24,25,26]. By employing MD simulations, researchers are able to delve deeply into the microscopic information and details of materials at the atomic and molecular levels [27,28]. A rising number of studies in recent years have employed MD simulations to elucidate the internal mechanisms of materials. For instance, Wu et al. [29] utilized MD to study the fracture behavior of kerogen, elucidating the fracture mechanisms within different types of kerogen. Abhiram et al. [30] conducted an MD study to investigate the effect of the degree of functionalization of single-walled CNTs on the fracture toughness of CNT/PMMA composites. The study provided a molecular-level explanation of the underlying mechanisms of crack propagation, crack arresting, and formation of new cracked surfaces. Chen et al. [31] conducted tensile simulations of phthalonitrile resins with the addition of toughened bodies using MD to elucidate the mechanism of toughening of phthalonitrile resins with BPSiPEN. The formation and expansion of free-volume pores in the material hinder the propagation of cracks, allowing more energy to be consumed during the fracture process and thus enhancing the fracture toughness of the material. Moreover, the efficacy of the MD simulation is corroborated by experimental evidence.

The research group has investigated the enhancement of helium gas barrier performance in Type IV hydrogen storage tank liner materials by incorporating modified montmorillonite into PA6 [32]. However, considering the toughness requirements for Type IV hydrogen storage tank liners, this study selects an HDPE with good toughness and PA6 with high gas barrier performance for blending. This study investigates the fracture toughness of HDPE/PA6 blends with varying ratios using MD simulations. A series of single-edge cracked specimens were simulated under tensile conditions, and uniaxial tensile loading of all crack-containing unit cells was carried out using the constant strain method. The fracture toughness of the composites was estimated by calculating the J-integral. Furthermore, to verify the reliability of the MD simulation results, we also tested some of the composites using the essential work of fracture (EWF) method.

## 2. Simulation Method

**Figure 1:**
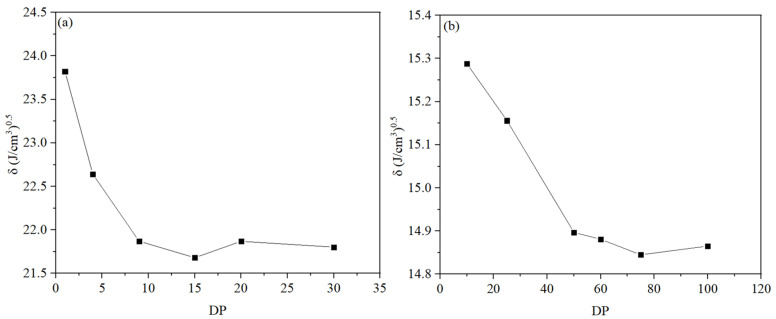
Solubility parameters versus degree of polymerization of (**a**) PA6 and (**b**) HDPE.

**Figure 2:**
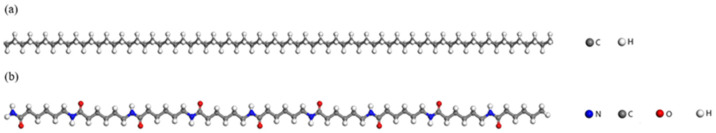
Schematic diagram of (**a**) HDPE and (**b**) PA6 single-chain modeling.

Considering the limitations of computational capacity, it is not feasible to simulate polymers with their real polymerization degrees. In this study, the selected degrees of polymerization (DP) were determined based on the stabilization trend of the solubility parameter [33]. The degrees of polymerization for polyamide 6 (PA6) were set to 1, 4, 9, 15, 20, and 30, while those for high-density polyethylene (HDPE) were set to 10, 25, 50, 60, 75, and 100. Amorphous unit cells were constructed using the Amorphous Cell module, followed by geometry optimization and a 100 ps molecular dynamics simulation in the NPT ensemble at 298 K. The variation in the solubility parameter with the degrees of polymerization for PA6 and HDPE is shown in Figure 1. Based on this analysis, the degrees of polymerization for HDPE and PA6 were ultimately selected as 75 and 9, respectively. The single-chain models are shown in Figure 2. Materials Studio 2017 [34] was used to construct all the models. The AC module was used to design six amorphous models with different HDPE/PA6 mass ratios of 10/0, 7/3, 6/4, 4/6, 3/7, and 0/10 for the MD simulations. The number of polymer chains in each model is shown in Table 1. The size ratio of all models in the tensile direction to the other two directions was set to 2:1:1, with each model containing approximately 19,000 atoms.

### 2.1. Tensile Simulation

All tensile simulations were conducted using LAMMPS (28 March 2023 version). The ReaxFF potential [35,36,37] was used to describe atomic interactions. ReaxFF is a bond-order-based potential that updates bond orders during the simulation, allowing for bond formation and breaking, which makes it particularly suitable for simulating fracture phenomena.

To obtain a stable model, the unit cell was first energy-minimized, with energy and force tolerances set to 10^−15^. The NVT system was then used to relax for 10 ps at 298 K to stabilize the system temperature at 298 K. To further bring the system to a steady state, a final NPT simulation was performed at 298 K and 0.1 MPa for 100 ps. The simulation used a timestep of 0.5 fs, with the damping coefficients for temperature and pressure set to 50 fs and 500 fs, respectively.

**Figure 3:**
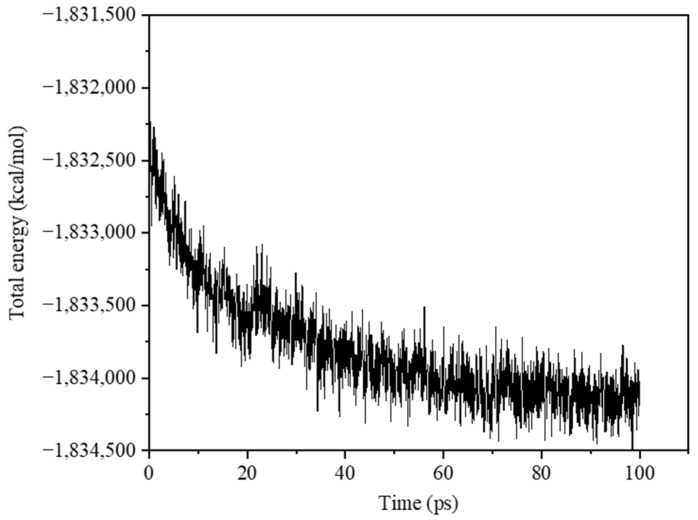
Curve of total energy change during relaxation.

Figure 3 demonstrates the variation in the total energy of PA6 during relaxation with simulation time; as the relaxation time increases, the total energy of the system tends to stabilize, which indicates that a stable structure is reached.

**Figure 4:**
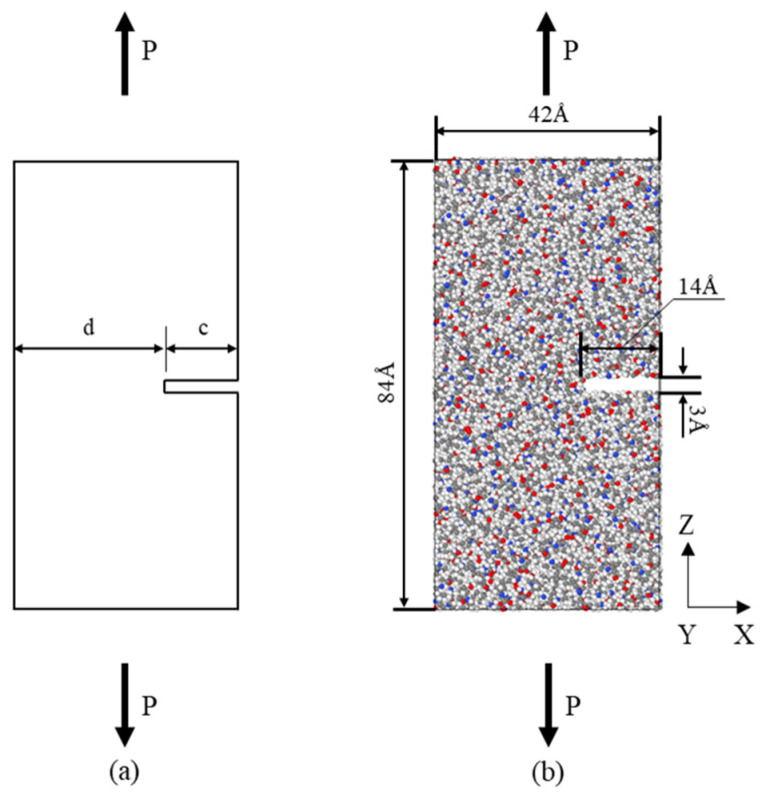
Single-edge notched tensile (**a**) specimen plates and (**b**) PA6 model.

To investigate the fracture properties of the material, an edge crack with a length of 14 Å and a width of 3 Å was created in the middle of the unit cell. Figure 4b shows the PA6 tensile model with a crack. The model was simulated in tension using the NPT system at a temperature of 298 K. To capture the Poisson effect, the pressure component of the model perpendicular to the loading direction was set to 0.1 MPa. The tensile rate was set to 0.0001 fs^−1^.

### 2.2. J-Integral Calculation

To further investigate the fracture behavior of the composite material, this paper calculates its fracture toughness using the J-integral method. The plastic contribution of the material during fracture is much higher than the elastic part. Therefore, the J-integral is calculated by ignoring the elastic part. For the specimen with a single-edge crack of thickness *B* in Figure 4a, the calculation of the J-integral is given by [30]:

(1) $$J=\frac{A_{P}}{d\times B}$$

where *A_P_* is the plastic area under the load–displacement curve during tensile loading, and *d* is the uncracked length of the plate.

### 2.3. Fracture Analysis

**Figure 5:**
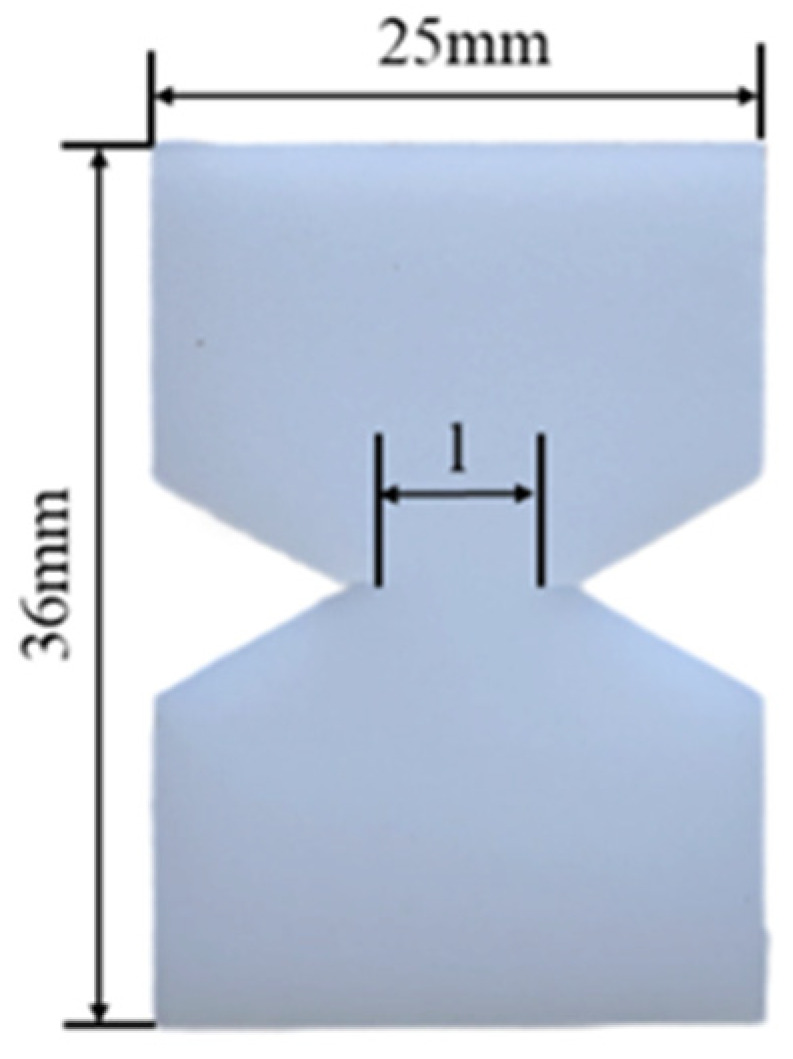
Double-edge notched tensile specimens.

The material’s fracture toughness was evaluated through the EWF method with a specimen size of 36 × 25 × 1 mm^3^ and tested by applying uniaxial tension to double-edge notched tensile (DENT) specimens, which are shown in Figure 5. The ligament length (l) was between 4 and 8 mm (5 ligament lengths). The cracks were prefabricated using a sharp blade perpendicular to the tensile loading direction. For each material, at least five specimens of each ligament length were tested. The tests recorded the load–displacement curves.

## 3. Results and Discussion

### 3.1. Fracture Toughness and Crack Extension of Materials

**Figure 6:**
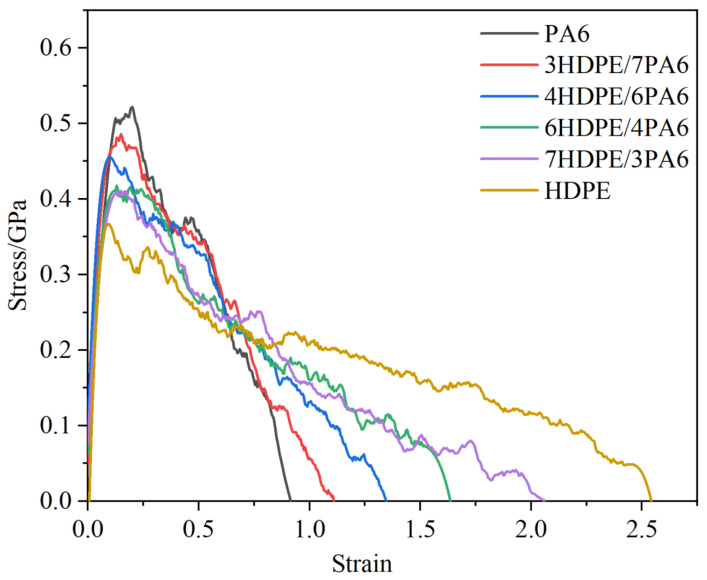
Stress–strain curves of crack-containing unit cells of HDPE/PA6 composites with different mass ratios.

To investigate the fracture properties of the HDPE/PA6 composites with different mass ratios, uniaxial tensile loading of crack-containing unit cells was performed using the constant strain method. The stress–strain curves for all composite materials are presented in Figure 6. The jagged shape of the curves in Figure 6 results from the combined effect of slip/tension and the breaking of covalent bonds [38]. As illustrated in Figure 6, the tensile strength of the composites exhibits an increase with the addition of PA6, whereas the elongation at break demonstrates an inverse trend. This result may be caused by the fact that the molecular chain of PA6 contains amide groups, which are able to form a large number of hydrogen bonds to make neighboring molecules form a strong intermolecular force, making the combination between molecular chains more tightly, thus enhancing the strength of the composites. However, the presence of hydrogen bonds also constrains the free deformation capacity of the molecular chain in response to external forces, which consequently reduces the elongation at break of the composite material. Table 2 presents the data regarding the strength and fracture parameters of the composites with varying ratios.

**Figure 7:**
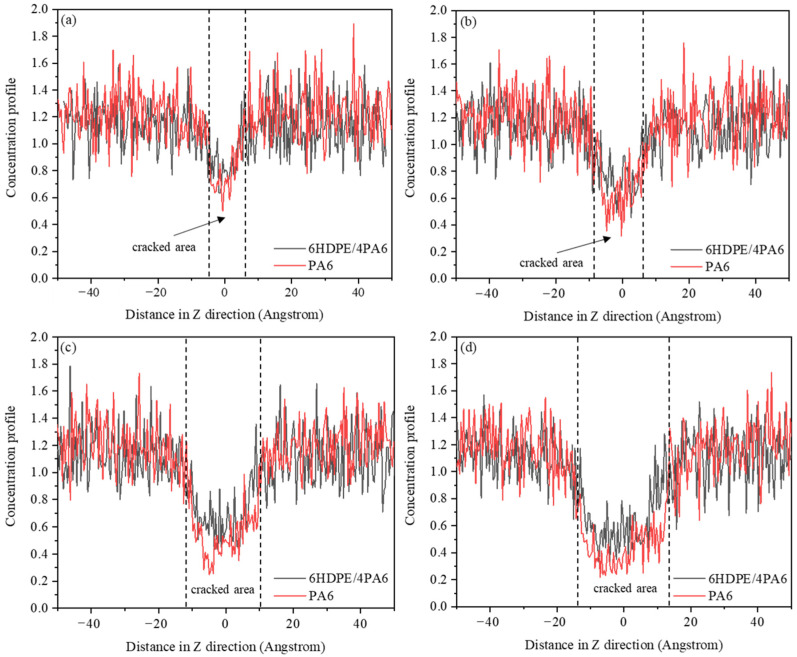
Concentration distribution of PA6 and 6HDPE/4PA6 at different strains: (**a**) 20% strain, (**b**) 30% strain, (**c**) 40% strain, and (**d**) 50% strain.

This paper presents a quantitative analysis of the fracture toughness of the composites using the J-integral method, with the fracture toughness values calculated from Equation (1). The specific numerical results are presented in Table 2. From these data, it can be observed that the fracture toughness of the composites increases gradually with the increase in HDPE content. The addition of HDPE enables the composites to undergo more plastic deformation and molecular chain slip, which effectively disperses the localized stresses and absorbs more energy, thereby slowing down the expansion rate of the cracks. To gain deeper insight into the toughening mechanism of the composites, a detailed analysis of the crack extension behavior of the PA6 and 6HDPE/4PA6 composites during the tensile process was conducted. Figure 7 shows the atomic concentration distribution of the crack-containing unit cells at 20%, 30%, 40%, and 50% strain for the two materials.

As shown in Figure 7, at 20% strain, the atomic concentration distribution of pure PA6 forms a distinct groove structure along the Z-axis, with an average concentration of 0.77 and a minimum concentration of 0.50. When the strain is increased to 30%, 40%, and 50%, the average concentration of the groove decreases to 0.67, 0.55, and 0.47, with reductions of 13%, 29%, and 39%, respectively. The minimum concentration in the groove also decreases from 0.50 to 0.32, 0.25, and 0.22, with reductions of 36%, 50%, and 56%, respectively. Meanwhile, the width of the groove gradually increases from 10 Å to 15 Å, 21 Å, and 28 Å. These changes indicate that as the strain increases, the atomic concentration in the PA6 matrix gradually decreases, the groove structure expands, and the material’s degree of damage intensifies.

**Figure 8:**
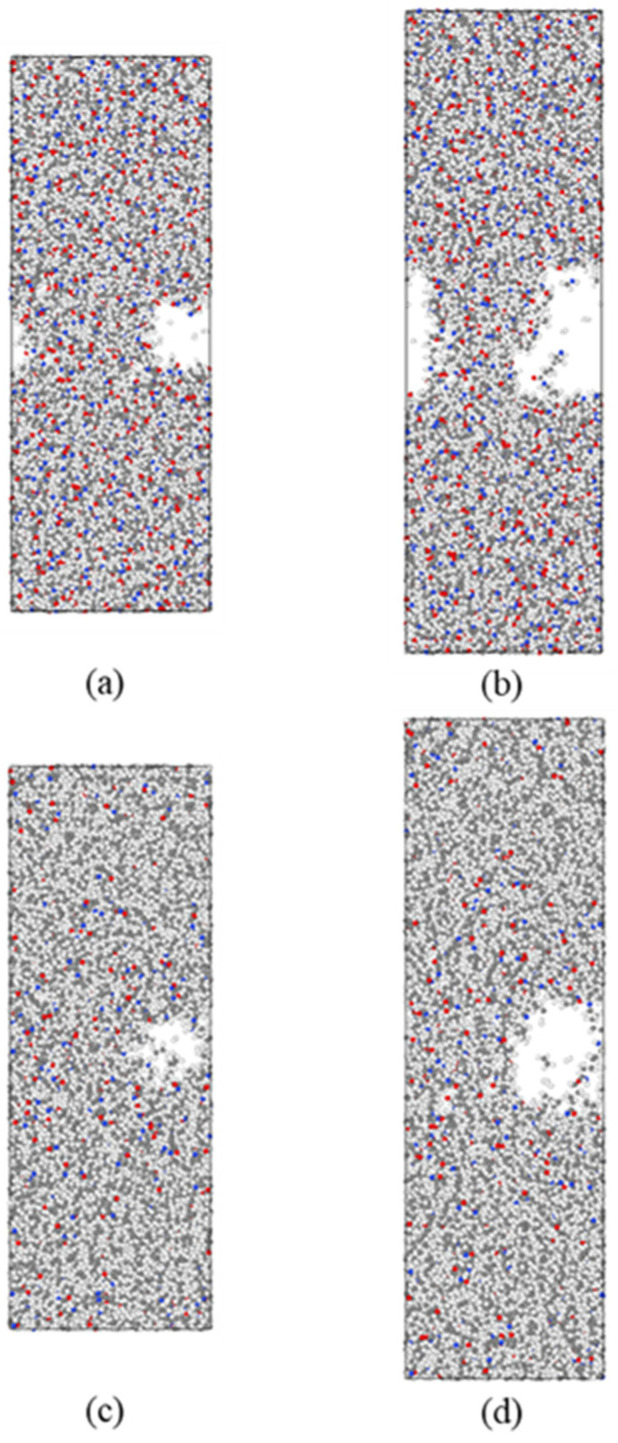
Snapshots of pure PA6 (**a**,**b**) and 6HDPE/4PA6 (**c**,**d**) at strains of 30% and 50%.

With the incorporation of HDPE, the cracking of the composites was effectively suppressed. At different strain levels (20%, 30%, 40%, and 50%), the average concentration of the groove in the 6HDPE/4PA6 composite is 0.81, 0.76, 0.68, and 0.56, respectively, which is approximately 5%, 13%, 24%, and 19% higher than that of the pure PA6 matrix. Moreover, under the same strain conditions, the minimum concentration of the groove is 0.63, 0.45, 0.42, and 0.31, which represents an increase of about 26%, 41%, 68%, and 41%, respectively, compared to the pure PA6 matrix. Additionally, the width of the groove is reduced, especially when the strain reaches 50%, where this change is particularly noticeable. Figure 8 shows snapshots of the pure PA6 and 6HDPE/4PA6 at strains of 30% and 50%. From the figure, it can be observed that as the strain increases, the presence of HDPE effectively enhances the structural stability of the composite material. A large number of atoms remain in the crack region, which helps to effectively suppress the crack propagation.

### 3.2. Experimental Test

#### 3.2.1. EWF Theory

**Figure 9:**
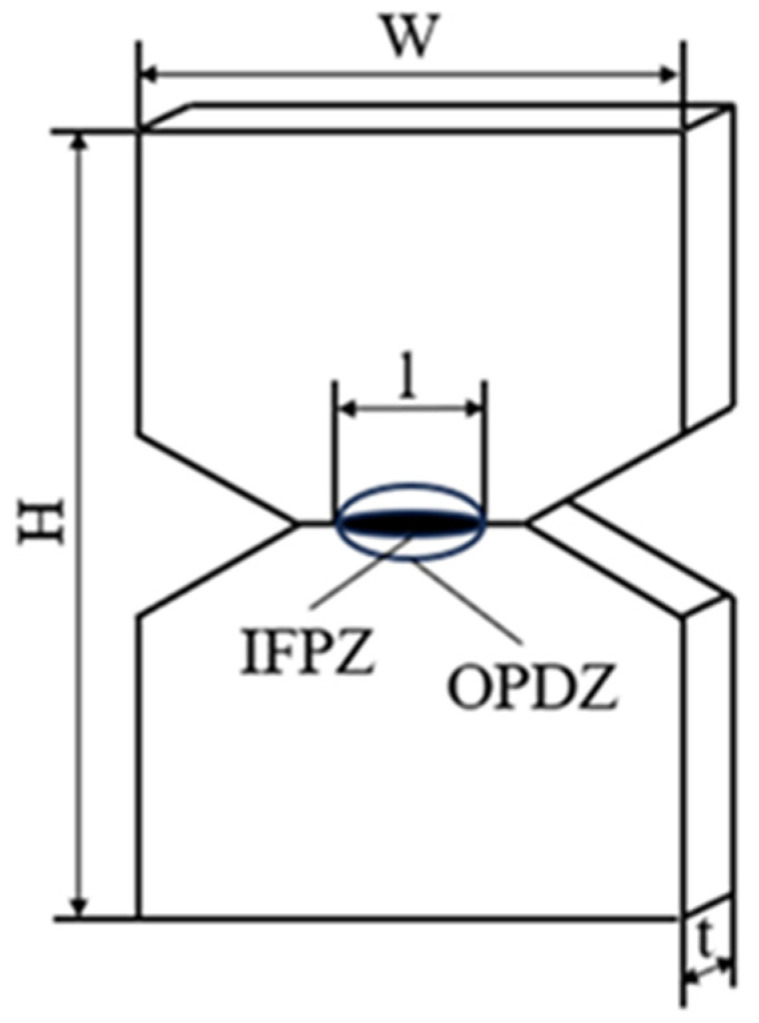
Schematic of DENT specimen.

In recent years, the EWF method proposed by Broberg [39] has been widely applied to assess the fracture toughness of polymer materials. The EWF method suggests that the overall fracture energy *W_f_* required to break a double-edge notched tensile (DENT) specimen can be divided into two components: the essential work of fracture *W_e_* and the non-essential work of fracture *W_p_*. Specifically, *W_e_* represents the energy dissipated in the inner fracture process zone (IFPZ), and *W_p_* represents the energy dissipated in the outer plastic deformation zone (OPDZ). Figure 9 illustrates a schematic of these two regions in a DENT specimen. In the figure, H is the specimen length, W is the specimen width, t is the specimen thickness, and l is the ligament length. The fracture work and its corresponding component relationships are described as follows:

(2) $$W_{f}=W_{e}+W_{p}$$

where *W_e_* is essentially a surface energy term, and *W_p_* is essentially a volumetric energy term. For a specimen with a fixed thickness (*t*), *W_e_* is proportional to the ligament length (*l*), and *W_p_* is proportional to *l*^2^. Therefore, Equation (2) can be written as:

(3) $$W_{f}=w_{e}lt+\beta w_{p}l^{2}t$$

(4) $$w_{f}=W_{f}/lt=w_{e}+\beta w_{p}l$$

where *w_e_* is the specific essential work of fracture, the work done per unit area of crack extension; *w_p_* is the specific non-essential work of fracture, which is also the plastic work per unit area; *β* is the shape factor of the plastic zone, which is only related to the shape of the specimen. From Equation (4), it can be seen that *w_f_* is linearly related to *l*. If DENT specimens are subjected to low stress, *w_f_* is the energy required to detach a unit area of the specimen. *W_f_* is obtained from the area under the load–displacement curve, and *W_f_* per unit area is *w_f_*. For a set of specimens with different ligament lengths made to fit the *w_f_*-*l* diagram, a straight line is obtained by linear regression, the intercept of the straight line extended to the y-axis is *w_e_*, and the slope is *βw_p_*.

#### 3.2.2. Load–Displacement Curve

**Figure 10:**
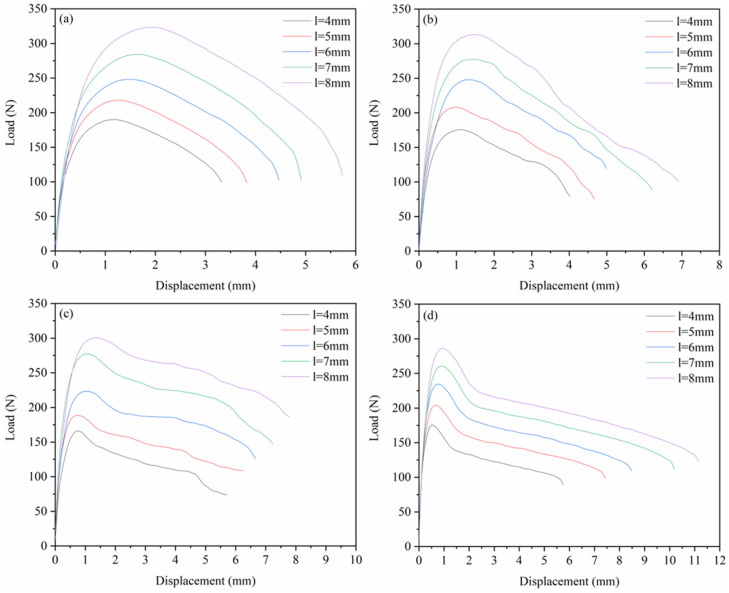
Load–displacement curves of the DENT specimens under the EWF test: (**a**) PA6, (**b**) 4HDPE/6PA6, (**c**) 7HDPE/3PA6, and (**d**) HDPE.

Figure 10 shows the load–displacement curves of the DENT specimens in the EWF test for four materials (PA6, 4HDPE/6PA6, 7HDPE/3PA6, and HDPE). From the figure, it can be observed that the maximum load of the load-displacement curves for all the materials shifts to the right and upward as the ligament length increases. This “self-similarity” is an important prerequisite for the validity of the EWF test results, as it ensures that, under the same testing conditions, the crack propagation process is independent of the ligament length [40,41].

Comparing the load–displacement curves of each material in Figure 10, it can be seen that the maximum load of the material decreases as the HDPE content increases. This phenomenon can be attributed to the lower strength of the HDPE, which leads to a reduction in the load-carrying capacity of the composite. However, the fracture displacement of the material increases, which can be attributed to the improved flexibility and reduced brittleness of the HDPE molecular chains under tensile loading. This enables the composite material to absorb more energy and undergo greater displacement under tension, thereby delaying crack propagation and enhancing its fracture toughness.

**Figure 11:**
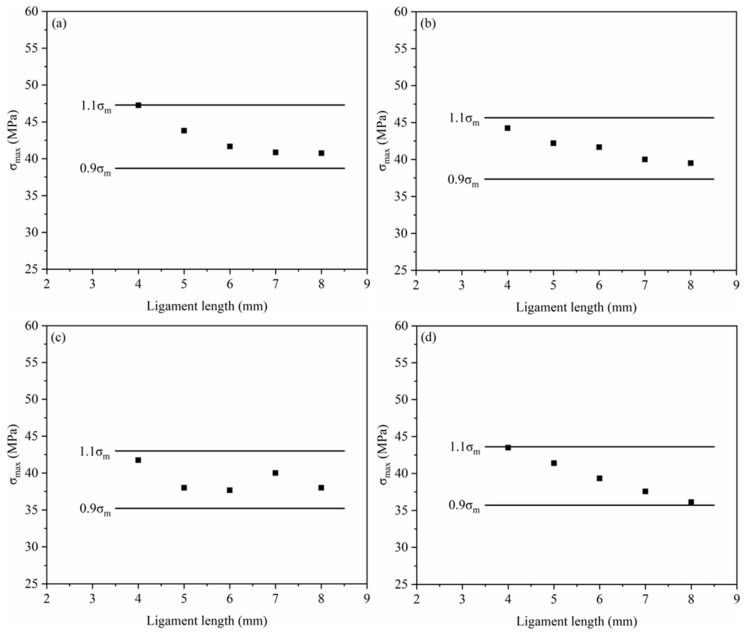
Sai-Hill guideline diagrams for (**a**) PA6, (**b**) 4HDPE/6PA6, (**c**) 7HDPE/3PA6, and (**d**) HDPE.

To verify the validity of the test results, the results were examined using the Sai-Hill criterion, as shown in Figure 11. The maximum static cross-sectional stresses (σ_max_ = F_max_/lt) for the different ligament lengths of each material in the figure lie in the range of 0.9σ_m_ to 1.1σ_m_ (where σ_m_ is the average of the maximum stresses). This result shows that the obtained data meet the requirements of the Sai-Hill criterion, thus validating the test results.

#### 3.2.3. EWF Parameters

**Figure 12:**
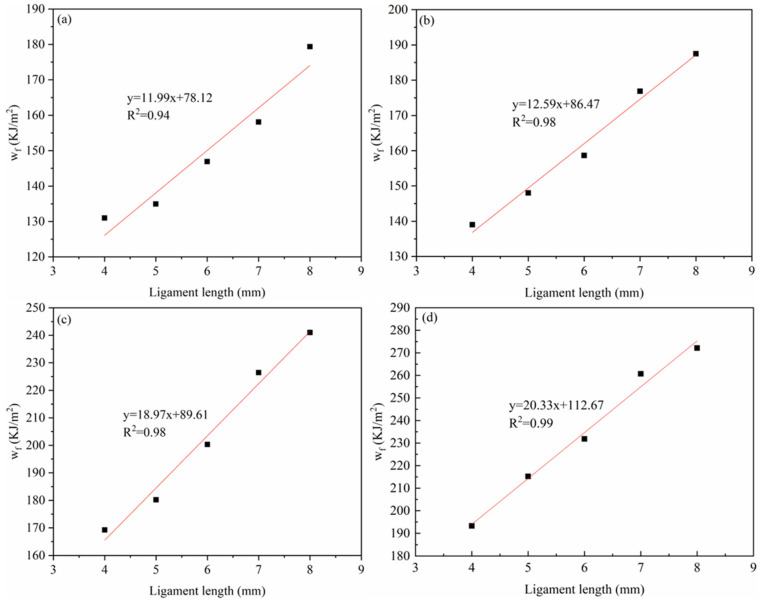
Scatter fit plots of w_f_ versus ligament length for (**a**) PA6, (**b**) 4HDPE/6PA6, (**c**) 7HDPE/3PA6, and (**d**) HDPE.

Figure 12 shows the scatter fit plots of w_f_ versus the ligament length for the four materials (PA6, 4HDPE/6PA6, 7HDPE/3PA6, and HDPE) in the EWF test. From the figure, it can be seen that the fitted lines for each material exhibit a good linear correlation, with the linear regression coefficient (R^2^) greater than 0.9, indicating a high degree of data fit. The corresponding numerical values are listed in Table 3.

In theory, *w_e_* is a material constant that depends solely on the specimen thickness. As an indicator for assessing a material’s fracture toughness, it is equivalent to the critical J-integral in assessing the material’s fracture toughness. The results in Table 3 show that the *w_e_* of the PA6/HDPE composites gradually increases with the increase in the HDPE content. This means that more energy is consumed in the internal tearing process region during the crack extension of the material, thus indicating an improvement in the fracture toughness of the material and an increase in the resistance to crack extension. The results are consistent with the changing trend of the simulation analysis, which further verifies the positive influence of the HDPE on the fracture properties of composites and provides a valuable reference for the design and optimization of materials.

## 4. Conclusions

This study employed MD simulations to investigate the effect of different mass ratios (10/0, 7/3, 6/4, 4/6, 3/7, and 0/10) of PA6/HDPE composites on their fracture toughness. The fracture toughness of the four materials (PA6, 4HDPE/6PA6, 7HDPE/3PA6, and HDPE) was calculated by using the EWF method. Our research shows the following:

1. With an increase in the HDPE content, the tensile strength of the composites gradually decreases, while the elongation at break and fracture toughness show gradual improvement trends. In the fracture process, the addition of HDPE can make the composite material undergo more plastic deformation and molecular chain slip, which effectively disperses the local stress, thus absorbing more energy and slowing down the expansion of the crack. The decrease in atomic concentration in the crack region is effectively slowed down, which enhances the resistance of the material to crack expansion.
2. The fracture toughness of the four materials was calculated by the EWF method. The results reveal that as the HDPE mass ratio increases, the w_e_ of the PA6/HDPE composite material also gradually improves. The trend observed in the simulation results was in agreement with the experimental results, verifying the effectiveness of the molecular dynamics simulation method in predicting the fracture toughness of materials.

While this study offers insights into the fracture toughness of PA6/HDPE composites, hydrogen permeability, a critical factor for hydrogen storage performance, was not examined. Future research should include hydrogen permeability tests to comprehensively assess these composites and determine the optimal configuration for storage applications, aiding in the selection of the most suitable materials.

## Figures and Tables

**Table 1:** Molecular chain composition in the simulation models.

Models	Number of HDPE Chains	Number of PA6 Chains
PA6	0	110
3HDPE/7PA6	15	72
4HDPE/6PA6	19	59
6HDPE/4PA6	27	37
7HDPE/3PA6	31	28
HDPE	43	0

**Table 2:** Strength and fracture parameters of HDPE/PA6 composites with different mass ratios.

Material	Tensile Strength (GPa)	Ultimate Strain (%)	J-Integral (N m^−1^)	Improvement in J (%)
PA6	0.522	0.915	3.035	Baseline
3HDPE/7PA6	0.486	1.110	3.096	2
4HDPE/6PA6	0.456	1.345	3.291	8
6HDPE/4PA6	0.418	1.635	3.441	13
7HDPE/3PA6	0.411	2.060	3.619	19
HDPE	0.367	2.540	4.436	46

**Table 3:** Specific essential work of fracture and linear regression coefficients of PA6/HDPE composites with different components.

Material	*w_e_* (kJ m^−2^)	R^2^	Improvement in *w_e_* (%)
PA6	78.12	0.94	Baseline
4HDPE/6PA6	86.47	0.98	11
7HDPE/3PA6	89.61	0.98	15
HDPE	112.67	0.99	44

## Data Availability

The original contributions presented in this study are included in the article. Further inquiries can be directed to the corresponding author.
